# A Rare Case of Nodal T-follicular Helper Cell Lymphoma That Developed Cutaneous Lesions With Numerous Epstein-Barr Virus-Positive T Cells

**DOI:** 10.7759/cureus.103130

**Published:** 2026-02-06

**Authors:** Osamu Okamoto, Yuzo Oyama, Kumiko Narahara, Kentaro Nagamatsu, Morishige Takeshita

**Affiliations:** 1 Division of Dermatology, Oita City Medical Association’s Almeida Memorial Hospital, Oita, JPN; 2 Department of Pathology, Oita University Faculty of Medicine, Yufu, JPN; 3 Department of Hematology, Japan Community Healthcare Organization Nankai Medical Center, Saiki, JPN; 4 Division of Hematology, Oita City Medical Association’s Almeida Memorial Hospital, Oita, JPN; 5 Division of Diagnostic Pathology, Saiseikai Yahata General Hospital, Kitakyushu, JPN

**Keywords:** aggressive, cd4-positive t cells, epstein-barr virus, nodal t-follicular helper cell lymphoma, skin lesion

## Abstract

We report a case of nodal T-follicular helper cell lymphoma (TFHL) in an elderly patient who developed multiple cutaneous lesions containing abundant Epstein-Barr virus (EBV)-positive neoplastic T cells. A pharyngeal mass and systemic lymphadenopathy were identified in a 78-year-old male. A histological and immunohistochemical review demonstrated diffuse and nodular infiltrates composed of small- to medium-sized atypical CD3-, CD4- and TFH cell marker-positive lymphoid cells, with scattered EBV-positive B cells and T cells. A diagnosis of nodal TFHL was made, but the patient refused chemotherapy. Indurated erythematous plaques and cutaneous nodules involving the trunk and arms developed one year and eight months after the onset of the lymph node and pharyngeal lesions. A histological examination of the skin nodule demonstrated diffuse infiltration of atypical small- to medium-sized lymphoid cells in the dermis and subcutis. Immunohistochemistry demonstrated that the atypical lymphoid cells were CD3- and CD4-positive and expressed TFH cell markers. Numerous EBV-positive cells were identified among the CD3- and CD4-positive atypical lymphoid cells. Skin lesions of EBV-positive nodal TFHL were diagnosed.

The clinical course was aggressive despite chemotherapy, and the patient developed overt leukemic changes and ultimately died of the disease. This is the first reported case of EBV-positive nodal TFHL showing cutaneous lesions demonstrating intense EBV infection in CD3- and CD4-positive lymphoid cells. The presence of EBV- and CD4-positive T cells in the initial biopsy specimens and the subsequent appearance of skin lesions with numerous EBV- and CD4-positive lymphoid cells may indicate accelerated EBV infection and an aggressive phenotypic transformation.

## Introduction

T-follicular helper (TFH) cells are T cells that express TFH markers such as CD10, BCL6, PD1, CXCL13, and ICOS [1]. Peripheral T cell lymphoma (PTCL) is a subtype of malignant lymphoma that originates from mature T cells or natural killer (NK) cells [1]. Among PTCLs, nodal TFH cell lymphoma (nTFHL) originates from TFH cells in the lymph nodes [1]. TFHL is classified into three types: angioimmunoblastic (AI) type, follicular (F) type, and not otherwise specified (NOS), in the current fifth edition of the WHO classification [2]. These nTFHLs are closely related to Epstein-Barr virus (EBV) [3,4], and the lymph nodes are the primary sites of involvement; however, extranodal lesions are sometimes observed [3,4]. In nTFHL-AI, the most well-defined type, lymph node lesions demonstrate proliferation of neoplastic TFH cells with B cells that are very often EBV-positive, while the T cells are not infected with EBV [3]. Skin lesions have been reported in nTFHL, particularly in nTFHL-AI, and are generally mild or subtle, with EBV detection in infiltrating lymphocytes being uncommon [3,5]. Therefore, nTFHL with extensive EBV infection of T cells in the lymph nodes and skin lesions is exceptionally rare. Our review of the relevant literature revealed only one report describing two cases of nTFHL with EBV-infected T cells in the lymph nodes, but lacking skin lesions [6].

We report a case of an elderly patient with nTFHL presenting initially as lymph node and pharyngeal lesions, followed by multiple cutaneous lesions containing numerous EBV- and CD4-positive neoplastic T cells. The patient eventually developed leukemic transformation and experienced a fatal, progressive clinical course. Furthermore, we were able to compare EBV infection patterns between the initial and subsequent lesions. EBV infection in T cells is extremely rare in nTFHL [6], and cases showing EBV-positive T cells in both early lymph node/pharyngeal lesions and subsequent cutaneous lesions with numerous EBV-positive neoplastic T cells have not been reported. This report enhances current understanding of nTFHL and may serve as a foundation for defining a distinct entity of nTFHL. It also provides a more practical perspective on the relationship between phenotype and prognosis in this unusual form of nTFHL.

## Case presentation

Clinical manifestation and histological examination

A 78-year-old male with a history of bleeding gastric ulcer noticed a pharyngeal tumor and systemic lymph node enlargement in July, in the first year of onset (one year and eight months before his first visit to our division). The patient had no history of malignant tumors and was not immunocompromised. He consulted a hospital in August, and biopsies of a cervical lymph node and the pharyngeal tumor were performed, and T-cell lymphoma was suspected. His initial lactose dehydrogenase (LDH) and soluble interleukin-2 receptor (sIL-2R) levels were 177 U/l and 4,750 U/ml, respectively.

**Figure 1:**
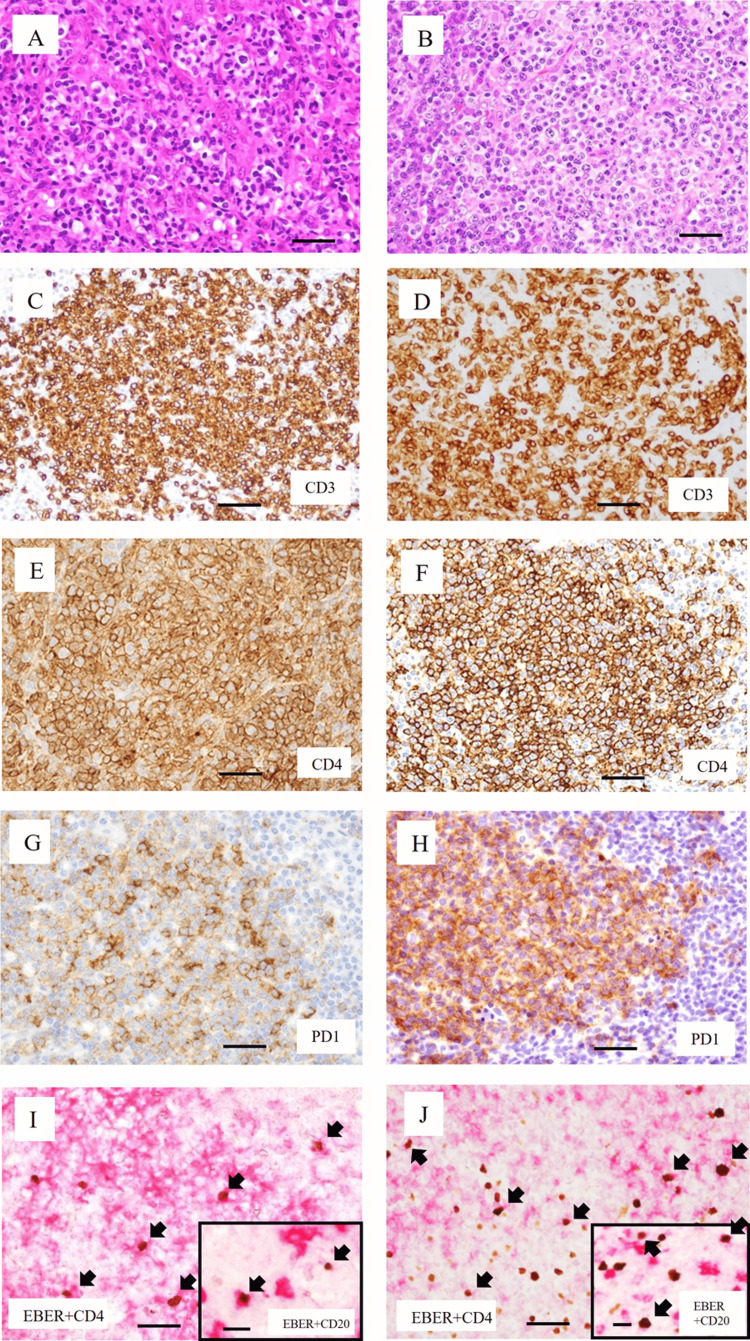
Histology and immunophenotypes of a pharyngeal tumor and a lymph node Histology and immunophenotypes of a pharyngeal tumor (A, C, E, G, I) and a lymph node (B, D, F, H, J) (×200) in August, in the first year of onset. A, B: H&E staining. Numerous lymphoid cells with clear cytoplasm are observed. C, D: CD3 staining. E, F: CD4 staining. G, H: PD1 staining. I, J: Double staining of EBER-ISH and CD4. Brown indicates positivity for EBER-ISH, and magenta indicates positivity for CD4. Arrows indicate representative positive reactions for EBER-ISH and CD4. In A-J, scale bars indicate 50 μm. Insets in I and J: Double staining of EBER-ISH and CD20 (×200). Brown indicates positivity for EBER-ISH, and magenta indicates positivity for CD20. Arrows indicate representative positive reactions for EBER-ISH and CD20. Scale bars in the inset indicate 10 μm

We reviewed the biopsied samples of the pharyngeal tumor (Figures 1A, 1C, 1E, 1G, 1I) and lymph node (Figures 1B, 1D, 1F, 1H, 1J) obtained in August, in the first year of onset. In both samples, diffuse and nodular infiltrates of small and medium-sized atypical lymphoid cells with clear cytoplasm were observed (Figures 1A, 1B). The immunohistochemical results are summarized in Table 1. As representative findings, similar nodular lesions of CD3- (Figures 1C, 1D), CD4- (Figures 1E, 1F), and TFH marker-positive cells (PD1: Figures 1G, 1H; BCL6: Table 1) were detected. However, the cytotoxic markers, CD8 and CD56, were negative (Table 1). *In situ* hybridization (ISH) revealed scattered EBV-positive small lymphoid cells, which were CD3- (not shown), CD4-, or CD20-positive by immunohistochemistry (Figure 1I, 1J, and insets). Based on these immunohistochemical profiles, the pharyngeal tumor and the lymph node lesion were diagnosed as nTFHL.

**Table 1:** Molecular marker profiles of the tumor cells of the pharyngeal tumor, lymph node (August in the first year of onset), and the skin (April in the third year of onset) “ +” indicates positive staining and “—” indicates negative staining; ND denotes not done. “ * ” indicates that overall staining was determined to be negative, but positive cells were focally accumulated or scattered

Markers	Pharyngeal tumor	Lymph node	Skin
CD3	+	+	+
CD4	+	+	+
CD5	+	+	+
CD8	-	-	-
CD30	-	-	-
CD10	+	+	-
BCL6	+	+	+
PD1	+	+	+
ICOS	ND	ND	+
CXCL13	ND	+	-
CD21	- *	- *	- *
CD56	-	-	-
TIA1	-	-	-
Granzyme B	-	-	-
CD20	- *	- *	- *
CD79a	ND	ND	-
FOXP3	ND	ND	-
MIB1	+	+	+
EBER-ISH	+	+	+
LMP1	ND	ND	-
EBNA2	ND	ND	-
CD25	weakly +		-
c-MYC	ND	ND	+
CCR4	ND	ND	-

**Figure 2:**
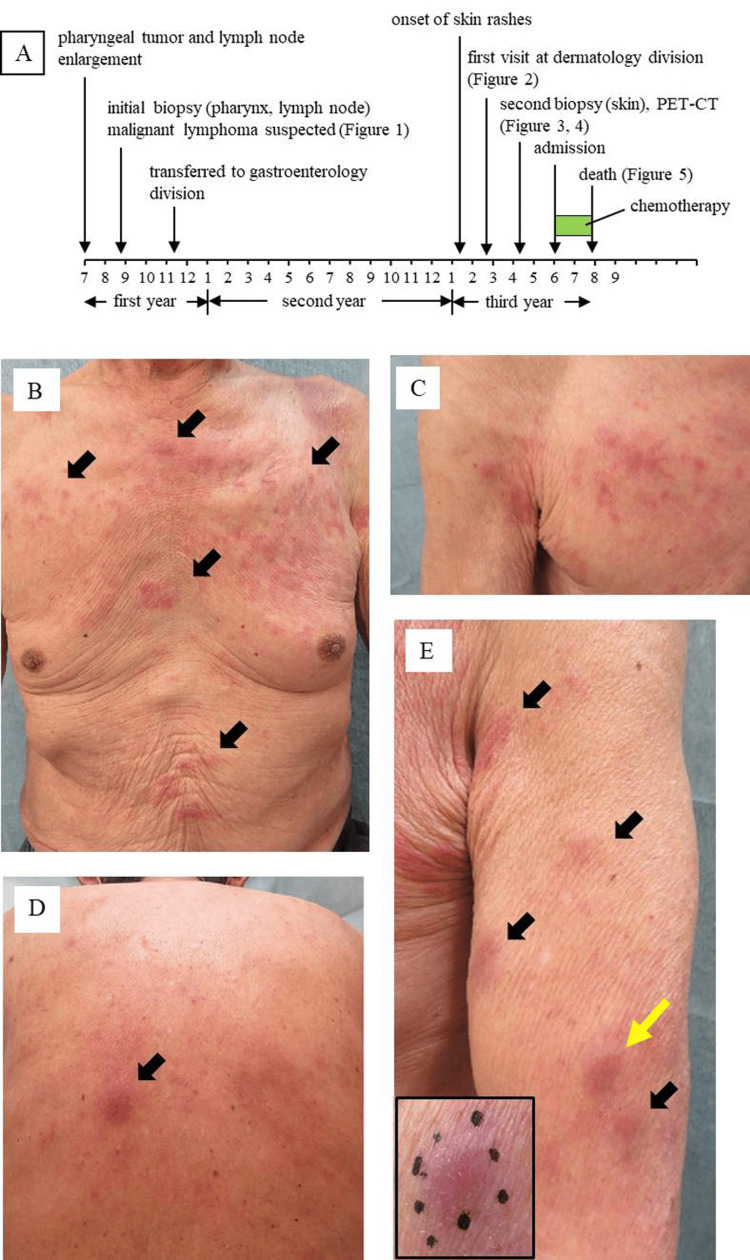
Clinical course and appearance A: The total clinical course of the current case. The horizontal bar indicates time. Time is expressed in month intervals (1-12). The years from onset are indicated at the bottom of the panel. B: Skin rashes on the anterior aspect of the trunk (arrows), C: rash on the right side of the chest, D: a tumor on the back (arrow), and E: tumors in the left upper arm (arrows). In panel E, the yellow arrow indicates a biopsied nodule. The inset shows a close-up view of a biopsied nodule

The clinical course of the patient is summarized in Figure 2A. Because the patient refused further examinations, the lesions were followed by simple observation for four months, and the follow-up was discontinued at the patient’s request. The patient was transferred to the gastroenterology division of our hospital in November of the first year of onset because of a bleeding gastric ulcer. At that time, the pharyngeal tumor had become undetectable, while the cervical lymph node enlargement persisted. His LDH level was normal, and his sIL-2R level was approximately 1,200 U/mL thereafter.

**Figure 3:**
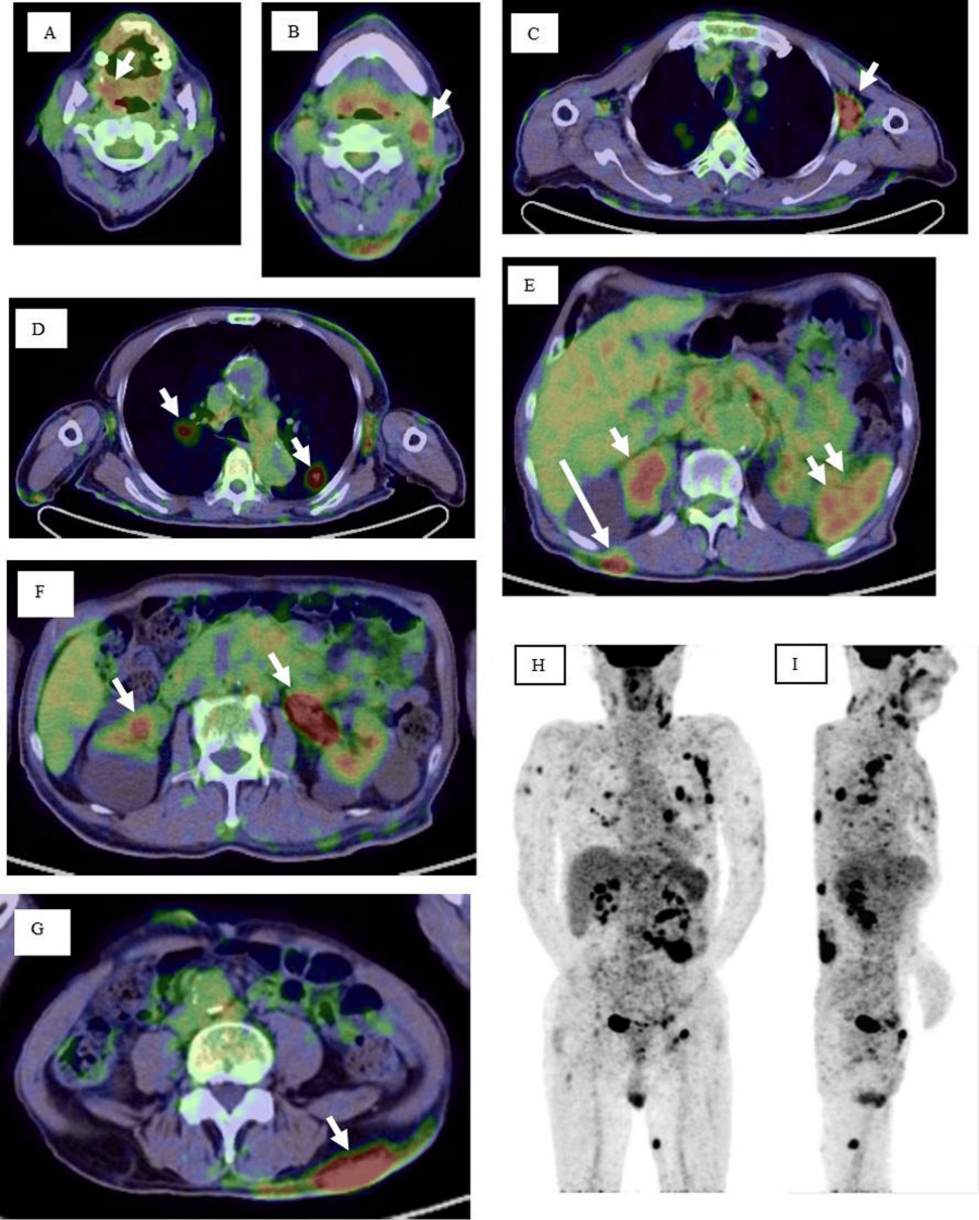
PET-CT performed in April in the third year of onset The abnormal uptake of ^18^F-fluorodeoxyglucose (FDG) is indicated by a red hue and pointed out by arrows. A: pharynx, B: lymph nodes of the neck, C: axilla, D: lung field, E and F: abdominal lymph nodes. In E, double arrows indicate the uptake in the spleen, and a long arrow indicates the surface of the back skin. G: back skin. H, I: maximum intensity projection in the early phase. Frontal view (H) and side view (I). The FDG uptake is shown in black. FDG accumulation in the brain and liver appears to be physiological PET-CT: positron emission tomography-computed tomography

Itchy erythema and multiple skin nodules appeared on the patient’s trunk in January of the third year of onset, and he consulted our division in February. Upon examination, his body temperature was approximately 37.5 ℃, and lymph nodes were palpable in the neck, axillary, and inguinal regions. Numerous indurated erythema and skin nodules were scattered on the chest (Figures 2B, 2C), upper back (Figure 2D), and upper arm (Figure 2E, arrows). The results of the blood examination are shown in Table 2. No abnormal lymphocytes were observed in the peripheral blood. EBV DNA was detected in the blood at a high titer (6.42 log IU/ml: 4.6-5.9 × 10^5 ^copies/μg), and anti-human T-lymphotropic virus (HTLV)-1 antibody was negative. Slight hypergammaglobulinemia and a high sIL-2R level (2,256 U/mL) were observed. PET-CT performed in April demonstrated abnormal uptake of 18F-fluorodeoxyglucose in the pharynx (Figure 3A, arrow), lymph nodes (arrows in Figures 3B, 3C, 3E, 3F), spleen (Figure 3E, double arrow), lung (Figure 3D, arrow), and the skin of the back (Figure 3E, long arrow; Figure 3G, arrow). The upper arms and the left upper leg also showed abnormal uptake (Figures 3H, 3I).

**Table 2:** Blood analysis data (February and July in the third year of onset) “ - ” indicates negative results. H and L denote abnormally high and low values, respectively. ND denotes not done

Items	Reference range	Value in February in the third year of onset	Value in July in the third year of onset
Temperature	36.0-37.0 ℃	37.5 (H)	37.5 (H)
Total protein	6.4-8.4 g/dl	6.9	ND
Albumin	3.6-5.2 g/dl	3.9	1.9 (L)
Albumin/globulin (A/G) ratio	1.3-2.0	1.3	ND
Total bilirubin	0.2-1.2 mg/dl	0.4	1.2
hemoglobin A1c	4.6-6.2%	5	ND
Urea nitrogen (UN)	6-22 mg/dl	25 (H)	44 (H)
Creatinine	0.6-1.1 mg/dl	1.39 (H)	2.75 (H)
Uric acid	2.5-7.0 mg/dl	6.3	4.8
Glucose	70-109 mg/dl	156 (H)	110 (H)
Aspartate aminotransferase (AST)	13-33 U/l	17	246 (H)
Alanine aminotransferase (ALT)	6-30 U/l	9	121 (H)
Lactose dehydrogenase (LDH)	106-211 IU/l	174	834 (H)
Llkaline phosphatase (ALP)	38-113 U/l	64	106
γ-glutamyl transpeptidase (γ-GTP)	16-73 U/l	14 (L)	134 (H)
Na	135-146 mEq/l	141	133 (L)
K	3.5-5.0 mEq/l	4.8	3.8
Cl	96-108 mEq/l	104	103
Ca	8.5-10.0 mg/dl	9.1	6.1 (L)
C-reactive protein (CRP)	0-0.23 mg/dl	0.18	13.2 (H)
Soluble interleukin-2 receptor (sIL-2R)	145-519 U/ml	2256 (H)	4345 (H)
Immunoglobulin G (IgG)	870-1,700 mg/dl	2056 (H)	ND
Immunoglobulin A (IgA)	110-410 mg/dl	280.7	ND
Immunoglobulin M (IgM)	35-220 mg/dl	66.7	ND
Anti-human T-lymphotropic virus (HTLV)-1/2 antibody	-	-	-
White blood cell (WBC)	3,900-9,800 /μl	5970	300 (L)
‧ Blast	0%	ND	0
‧ Promyelocyte	0%	ND	0
‧ Myelocyte	0%	ND	0
‧ Metamyelocyte	0%	ND	0
‧ Stab cell	0-6%	ND	0
‧ Segmented neutrophil	32-73%	56.1	0 (L)
‧ Lymphocyte	18-59%	34.5	36
‧ Monocyte	0-8%	6.7	1
‧ Eosinophil	0-7%	2	0
‧ Basophil	0-2%	0.7	0
‧ Atypical lymphocyte	0-2%	0	0
‧ Abnormal lymphocyte	0%	0	63 (H)
Red blood cell (RBC)	4.2-5.7 ×10^6^/μl	4.28	2.5 (L)
Hemoglobin	13.5-17.6 g/dl	14.1	7.9 (L)
Hematocrit	39.8-51.8%	43.4	22.5 (L)
Mean cellular volume (MCV)	82.7-101.6 fl	101.4	90
Mean corpuscular hemoglobin (MCH)	28-34.6 pg	32.9	31.6
Mean corpuscular hemoglobin concentration (MCHC)	31.6-36.6%	32.5	35.1
Platelet	131-362 ×10^3^/μl	160	12 (L)
Epstein-Barr virus (EBV)-DNA	(-) log IU/ml	6.42 (H)	ND

**Figure 4:**
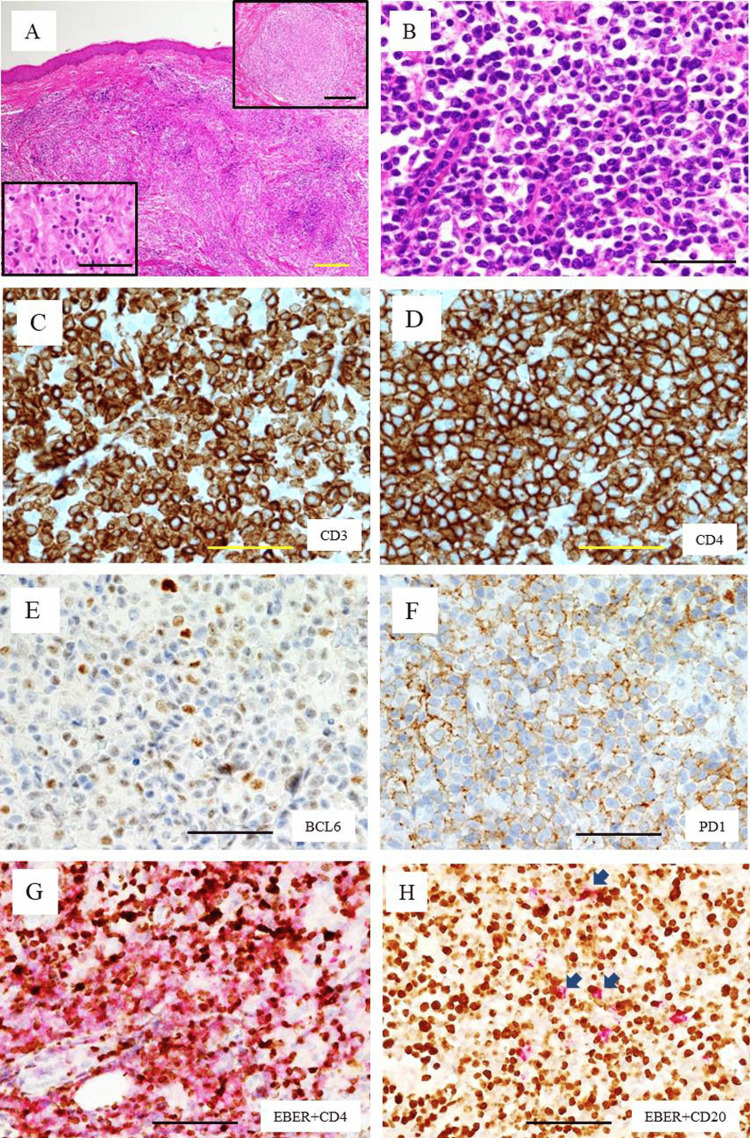
Histology and immunophenotypes of the skin tumor A, B: H&E staining. A: Low-power view (×40). Diffuse infiltration of lymphoid cells and histiocytes is observed in the dermis. Scale bar indicates 200 μm. Upper inset: clearly-demarcated granulomatous reaction (×40). Scale bar indicates 200 μm. Lower inset: close-up view of epithelioid cells and small lymphoid cells (×400). Scale bar indicates 50 μm. B: High power-view of the infiltrating neoplastic cells. Numerous small- to medium-sized lymphoid cells with clear cytoplasm are observed (×400). Scale bar indicates 50 μm. C-F: Immunostaining (×400). C: CD3, D: CD4, E: BCL6, F: PD1, G: double staining of EBER-ISH and CD4 (×400). Brown indicates positivity for EBER-ISH, and magenta indicates positivity for CD4. Note that numerous EBER-positive cells are positive for CD4. H: Double staining for EBER-ISH and CD20. Brown indicates positivity for EBER-ISH, and magenta indicates positivity for CD20 (×400). Arrows indicate EBV- and CD20-positive cells. In G and H, scale bars indicate 50 μm

A skin biopsy of a nodule on the arm (Figure 2E, yellow arrow and inset) was performed in April in the third year of onset, and H&E staining showed diffuse infiltration of atypical cells with granulomatous reactions in the dermis (Figures 4A, upper inset), but no epidermotropism (Figure 4A). The granulomatous reaction was composed of epithelioid cells and small lymphoid cells (Figure 4A, lower inset). The neoplastic cells were small- to medium-sized atypical lymphoid cells (Figure 4B). The results of immunohistochemistry are shown in Figure 4 and Table 1. The atypical lymphoid cells were diffusely positive for CD3 (Figure 4C), CD4 (Figure 4D), TFH cell markers BCL6 (Figure 4E), PD1 (Figure 4F), ICOS, and MIB1; however, atypical cells were negative for CD8, CD10, CD20, CD56, and cytotoxic molecules (CMs) (TIA-1 and granzyme B) (Table 1). Numerous EBV-positive cells were present in the infiltrate by ISH, many of which were found to be CD3- and CD4-positive by immunohistochemistry (Figure 4G). A small number of CD20-positive cells were scattered, and a few were positive for EBV (Figure 4H). Southern blotting analysis detected rearranged bands of *TCRCB1 *genes (not shown). We confirmed the histological diagnosis as a cutaneous lesion of nTFHL with numerous EBV-positive neoplastic T cells.

Treatments and clinical course

**Figure 5:**
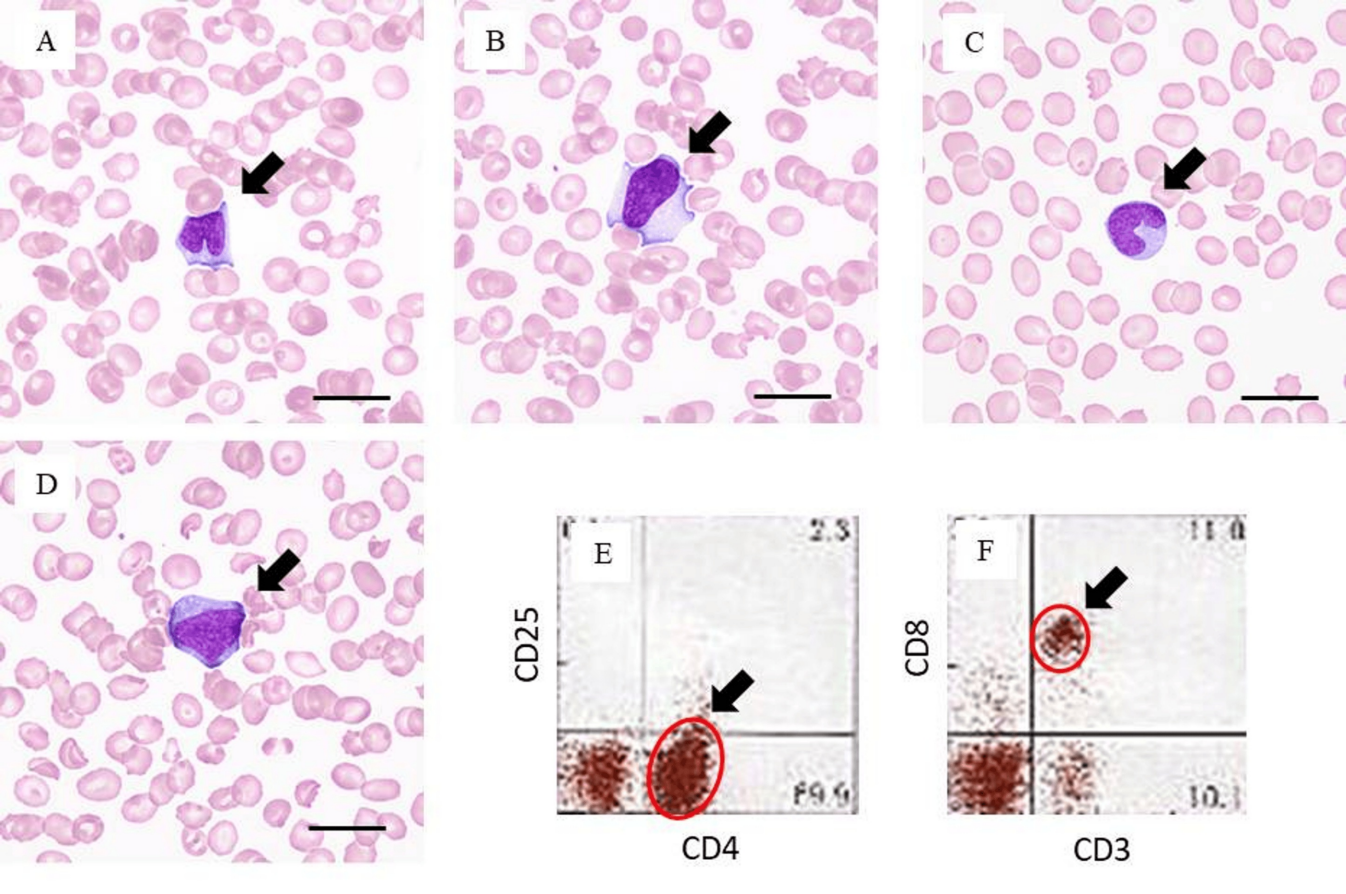
Blood examinations in the late stage (July in the third year of onset). A-D: Abnormal lymphocytes in the peripheral blood (×1,000). Note the bizarrely shaped lymphoid cells with irregularly shaped nuclei (arrow). Scale bars indicate 10 μm. E, F: Flow cytometry data. The positions of CD4- and CD8-positive cells are indicated by circles and arrows

Two courses of THP-CP-VDS therapy (pirarubicin 50 mg/m^2^, cyclophosphamide 750 mg/m^2^, prednisolone 90 mg/body, vindesine sulfate 3 mg/m^2^) were administered from June in the third year of onset (Figure 2A), and the lymph nodes decreased in size. However, leukocytopenia with abnormal lymphocytes in the peripheral blood appeared in June (Figures 5A, 5B, 5C, 5D), followed by facial cellulitis and pulmonary mycosis. Leukocytopenia progressed, and the WBC count fell to 300/μl with 63% abnormal lymphocytes. A diagnosis of leukemic change of nTFHL was made. The final laboratory data in July are summarized in Table 2. High CRP level, and hepatic and renal dysfunction were marked. The final sIL-2R was 4,345 U/ml. CD45 gating of flow cytometry demonstrated that 72% of the lymphocytes/lymphoid cells were CD4-positive, while only 14% were CD8-positive (Figures 5E, 5F), indicating similarity to the skin lesions. The patient died of disseminated intravascular coagulation and multi-organ failure. The total clinical course was two years and two months after the appearance of the pharyngeal tumor and lymph node enlargement (Figure 2A), demonstrating a progressive and aggressive course.

## Discussion

The current case of nTFHL is exceptional in that the patient demonstrated EBV- and CD4-positive neoplastic T cells in the lymph node and pharyngeal lesions, and the subsequent cutaneous lesion showed numerous EBV- and CD4-positive neoplastic T cells. Relevant nTFHL cases showing T cell predominant EBV infection in skin lesions have not been reported. The current case is also important in that the EBV infection status of the initial lymph node and pharyngeal lesions, which showed sparse EBV-positive T cells, and the advanced skin lesions, which showed numerous EBV-positive neoplastic T cells, could be compared.

EBV predominantly affects B cells and sometimes causes infectious mononucleosis; however, in most cases, strong host immune reactions involving cytotoxic T cells and NK cells are induced [7]. Consequently, infected B cells are eliminated, and the remaining EBV is maintained under immune control [7]. NK/T cells are also occasionally infected and eliminated, but in rare cases this elimination fails, probably due to dysfunction of cytotoxic T cells [8], and the disease may progress to EBV-related lymphoproliferative disorders (LPDs). The elimination of infected cells is heterogeneous because the causative cell types depend on the subtype of EBV-related LPDs [9,10]; however, the mechanisms by which specific cell types are selected and allowed to proliferate in EBV-related LPDs remain unclear.

EBV infection in infiltrating non-neoplastic B cells is frequently found in lymph node lesions of nTFHL-AI type [3,4]. In contrast, EBV infection in T cells is rarely seen in nTFHL, and even when present, the number of infected B cells usually predominates [11]. Wang et al. reported 23 cases of CD4- and TFH-positive primary cutaneous TFHL [12]. Six of the 23 cases had EBV-positive B cells, three developed EBV-positive diffuse large B cell lymphoma, one developed EBV-positive B-LPD, and two patients died of the disease at 12 and 48 months [12]. Zhang et al. reported two elderly EBV-positive nTFHL patients, one of whom presented with numerous EBV- and CD3-positive neoplastic cells in the lymph node and EBV-positive non-neoplastic T cells on double staining. Another patient presented with CD3-, CD4-, and TIA-1-positive neoplastic TFH cells in the lymph nodes [6]. These two patients developed lymph node and internal organ lesions, while skin rashes were not observed [6]. Chiang et al. also reported two elderly patients with primary cutaneous EBV-positive TFHL [13]. The phenotypes of the cases reported by Zhang and Chiang are similar to those of the current case, in that EBV infection was predominantly found in neoplastic T cells. Taken together, EBV-positive neoplastic T cells are rarely found in TFHLs, and EBV-infected TFH cells are suggested to induce neoplastic transformation into nTFHL, leading to the development of skin lesions and leukemic change, and ultimately resulting in lethal clinical courses.

One interesting point in the current case was that an identical immunophenotype of EBV-positive lymphoid cells had already been observed in lymph node and pharyngeal tumor samples taken one year and eight months before the appearance of the skin rashes, and the number of EBV-positive neoplastic T cells increased in the skin lesions as the disease progressed. No previous studies have examined EBV infection profiles in detail during the entire clinical course of the disease. Therefore, this report provides important information about the progression of EBV infection in T and B cells of nTFHL.

Another notable feature of the current case was that the disease was ultimately associated with leukemic change. Because 63% of the lymphocyte population was composed of abnormal lymphocytes, and the majority of the lymphocyte population was identified as CD4-positive T cells by flow cytometry, the phenotype of the neoplastic cells was consistent with that observed in the skin. The four relevant EBV-positive nTFHL cases mentioned above did not show leukemic changes [6,13]. Although bone marrow invasion and hepatosplenic invasion are frequently found in cases of systemic and nodal EBV-positive T-LPD, leukemic changes have rarely been reported or studied in detail. A cumulative report on the TFHL-AI type demonstrated peripheral blood involvement by neoplastic cells to various extents; however, in the majority of cases, the involvement was limited to up to 20% of the lymphocytic population [14]. The current case presented with overt leukemic changes, with a maximum of approximately 60% abnormal CD4-positive T cells. The unusual immunophenotype in the current case may have contributed to the leukemic transformation.

EBV-positive nodal NK/T-cell lymphoma (nNKTCL), extranodal NKTCL, and aggressive NK cell leukemia are representative EBV-positive malignant lymphomas [9]. These phenotypes consist of CM-positive T cells or CD56-positive NK cells, and the patients show a progressive clinical course [9,15]. Because they are well-recognized EBV-positive lymphomas, they are usually included in the differential diagnosis of other EBV-positive lymphoma phenotypes. Among them, EBV-positive nNKTCL is a primary diagnostic consideration [9]. EBV-positive nNKTCL predominantly affects the systemic lymph nodes, with occasional extranodal lesions in the liver, spleen, skin, gastrointestinal tract, and bone marrow. It usually demonstrates CD3- and CD8-positive and CD4- and CD56-negative neoplastic large T cells, and in rare cases, the neoplastic cells are CD4-positive [16]. However, neoplastic cells in nNKTCL are consistently positive for both EBV and CMs [9,15]. In the current case, because we did not perform a lymph node examination in the late stage of the disease, a diagnosis of nNKTCL could not be completely excluded for the lymph node lesions. However, the initial lymph node lesion was diagnosed as nTFHL; the lesions persisted until death, and the late-stage skin lesion was nTFHL. Based on the clinical course, it is considered likely that the lymph node lesions in the late stage also corresponded to nTFHL.

The THP-CP-VDS regimen is a modified version of the CHOP (cyclophosphamide, doxorubicin hydrochloride, vincristine, prednisolone) regimen, which is the first-choice therapy for many PTCLs [17]. The THP-CP-VDS regimen is often preferred in elderly patients [17]. Chiang et al. used the COP (cyclophosphamide, vincristine, and prednisolone) regimen for case 1 and the SMILE (dexamethasone, methotrexate, ifosfamide, L-asparaginase, and etoposide) regimen for case 2 [13], while Zhang et al. used the CHOP (cyclophosphamide, doxorubicin, vincristine, and prednisolone) regimen for cases 1 and 2, both of which resulted in fatal outcomes [6]. The current case did not respond to THP-CP-VDS therapy, ultimately developed leukemic change, and died before alternative regimens could be considered. Therefore, nTFHL is recognized as having an aggressive phenotype.

## Conclusions

The current case presented with nTFHL and subsequent development of cutaneous lesions containing numerous EBV- and CD4-positive neoplastic cells, and it demonstrated a rapidly progressive clinical course. Accelerated EBV infection of neoplastic T cells appeared to be associated with aggressive phenotypic conversion; however, the underlying mechanisms cannot be elucidated from a single case. Further insight will require the accumulation of additional cases. The current case was diagnosed as nTFHL, and a rare phenotype was identified by examining EBV infection profiles. However, some cases may be classified as nTFHL-AI or nTFHL-NOS. Therefore, careful examination of EBV infection may provide an opportunity to identify unusual phenotypes. The accumulation of relevant cases will allow a more practical understanding of the relationship between phenotype and prognosis in this unusual nTFHL.
